# Community engagement for the Voluntary Medical Male Circumcision (VMMC) program: an analysis of key stakeholder roles to promote a sustainable program in Zambia

**DOI:** 10.12688/gatesopenres.13587.1

**Published:** 2022-04-22

**Authors:** Joseph M. Zulu, Trevor Mwamba, Alyssa Rosen, Tulani Francis L. Matenga, Joseph Mulanda, Mutale Kaimba, Masitano Chilembo, Madaliso Silondwa, Royd L. Kamboyi, Sylvia Chila Simwanza, George Sichone, Malizgani Paul Chavula

**Affiliations:** 1 Department of Health Promotion and Education, School of Public Health, University of Zambia, PO Box 50110, Lusaka, Zambia, University of Zambia, Lusaka, 10101, Zambia; 2 Clinton Health Access Initiative, Clinton Health Access Initiative, Lusaka, 10101, Zambia; 3 Ministry of Health, Ministry of Health, Lusaka, Lusaka, 10101, Zambia

**Keywords:** Community engagement, voluntary medical male circumcision, HIV prevention, stakeholder analysis, power, roles, strategies, Zambia

## Abstract

**Background:** Within the Voluntary Medical Male Circumcision (VMMC) programme, community engagement has been central in facilitating the acceptance of VMMC, especially in non-circumcising communities. We used the case of the development of community engagement plans for sustainability of VMMC in Zambia to illustrate diversity of stakeholders, their power, roles, and strategies in community engagement.

**Methods:** Data were collected using document review, in-depth interviews (n=35) and focus group discussions (n=35) with community stakeholders, health workers, health centre committees, counsellors, teachers, community volunteers and parents/caregivers. Data were analysed using thematic analysis. The analysis was guided by the power and interest model.

**Results:** Differences were noted between the rural and urban sites in terms of power/influence and interest rating of community stakeholders who could be involved in the sustainability phase of the VMMC response in Zambia. For example, in the urban setting, neighbourhood health committees (NHCs), health workers, leaders of clubs, community health workers (CHWs), radio, television and social media platforms were ranked highest. From this list, social media and television platforms were not highly ranked in rural areas. Some stakeholders had more sources of power than others. Forms or sources of power included technical expertise, local authority, financial resources, collective action (action through schools, churches, media platforms, other community spaces), and relational power.   Key roles and strategies included strengthening and broadening local coordination systems, enhancing community involvement, promoting community-led monitoring and evaluation, through the use of locally recognised communication spaces and channels, facilitating ownership of VMMC, and improving local accountability processes in VMMC activities.

**Conclusions:** By consulting with the most relevant stakeholders, and considering community needs in programme development, the VMMC programme may be able to leverage the community structures and systems to reduce long term demand generation costs for VMMC and increase the acceptability and frequency of male circumcision.

## List of abbreviations

CHAs: Community Health Assistants;

CHWs: Community Health Workers;

FGD: Focus Group Discussion;

HIV: Human Immunodeficiency Virus;

KII: Key Informant Interview;

NHCs: Neighbourhood Health Committees;

VMMC: Voluntary Medical Male circumcision;

WDC: Ward Development Committee 

## Introduction

Human immunodeficiency virus (HIV) remains a global reproductive health issue and the largest cause of disease burden in sub-Saharan Africa ( Cork *et al*., 2020). In 2017, it was approximated that 25.9 million people were living with HIV in sub-Saharan Africa, with over one million newly infected in that year ( James *et al*., 2018). Many countries in Eastern and Southern Africa are especially severely affected by the pandemic, with a general population prevalence ranging from 5% in Tanzania to 27% in eSwatini (formerly Swaziland) ( Bailey *et al*., 2008). The HIV epidemic in Zambia is generalised, with heterosexual sex being the primary mode of transmission( ZDHS, 2018). Factors in the transmission of HIV in Zambia include multiple and concurrent partnerships (2% of women and 15% of men aged 15–49 reported having two or more sexual partners in the 12 months prior to the Zambia Demographic Health Survey - ZDHS), low and inconsistent condom use (35% of women and 54% of men reported using a condom during their last sexual intercourse with a non-marital or non-cohabiting partner-ZDHS), low coverage of medical male circumcision (32% of men aged 15–49 are circumcised-ZDHS), and mother-to-child transmission. The 2018 ZDHS estimates an HIV prevalence of 11% among men and women aged 15– 59 years ( ZDHS, 2018). The 2016 Zambia Population Based Impact Assessment Study ( Ministry of Health, 2019) earlier reported that among young adults (20–24 years of age), HIV prevalence was four times higher among women (8.3%) than among men (2.0%).

Voluntary medical male circumcision (VMMC), largely defined as the complete surgical removal of the foreskin from the penis, has been proven to be an effective intervention to reduce the risk of HIV transmission ( WHO, 2007). Prevention of HIV through community health strengthening, primary healthcare and increased universal health coverage is on the global agenda ( Bulstra *et al*., 2020). In 2007, the WHO and the joint United Nations Programme on HIV/AIDS (UNAIDS) recommended that VMMC should be considered an important intervention for HIV prevention in settings with high HIV and low circumcision prevalence ( WHO, 2007). This is in line with the UNAIDS fast-track strategy that explicitly calls for an end to the pandemic by 2030 ( Bulstra *et al*., 2020). VMMC is now implemented globally as part of the broader HIV prevention package in order to reduce HIV incidence and recommended by WHO as a high-impact cost-effective intervention particularly in settings with high HIV prevalence and low levels of male circumcision ( Odoch *et al*., 2015).

VMMC is a highly effective one-time intervention for preventing HIV transmission from females to males by approximately 60% for life ( Bailey *et al*., 2008) . Low and middle income countries like Zambia have reported significant success in scaling up VMMC programme service delivery in recent years as over 3 million men have been circumcised in 9 years between 2010–2019 ( Bulstra *et al*., 2020). The country achieved the VMMC National Operational Plan 2016–2020 target of 1.98 million men circumcised before the year 2020 ended ( Health, 2020). Furthermore, Zambia achieved over 100% of the annual VMMC target in 2018 and 2019 ( Health, 2020). While there been progress in the uptake of VMMC services, there are disparities between ages and locations in Zambia. The prevalence of men aged 15–49 who are circumcised increased from 13% in 2007 compared to 22% in 2013–14 and 32% in 2018 with highest being those among the age 20–24 (39%) and lowest among those aged 40–49 (23%). They were more men who were circumcised in the urban area (40%) compared with only 25% of in rural areas. Of those 32% percent of men in urban areas were circumcised by a health worker, compared with only 19% of men in rural areas ( Agency, 2020).

Global evidence studies also suggest the significance of sustaining VMMC interventions ( Odoch *et al*., 2015). Studies indicate that a sustainable VMMC programme is the “one whose local stakeholders maintain high circumcision prevalence after the initial scale-up—generally by incorporating either early infant male circumcision (EIMC), early adolescent male circumcision (EAMC), or both, into routine new-born and adolescent service delivery systems” (6, 7).

Given the burden of the HIV and impact of VMMC on averting new infections, the Ministry of Health in Zambia launched the Transition and Sustainability Plan for the VMMC Programme in 2019. The rationale of this plan was to enhance progress towards epidemic control by 2020 and contribute to eliminating new HIV/AIDS infections in Zambia by 2030. To ensure effective and sustainable demand generation for these targeted populations as well as the general population, it is essential that communities are adequately and intentionally engaged. However, implementation of the Transition and Sustainability Plan has been limited so far, and in part due to the lack of a community engagement plan. Primarily, the engagement plan is vital to increasing community level entry points, canvassing demand for services, and ensuring that VMMC becomes a norm at community level. In addition, the engagement plans are also necessary to promote ownership of VMMC, capacity building of local stakeholders and use of local resources through sharing of tasks ( Njeuhmeli *et al*., 2011; Njeuhmeli *et al*., 2014). The plans are also key to promoting participation of the local stakeholders in the implementation process of VMMC activities, including serving as the broader HIV prevention landscape by strengthening the local HIV coordination systems ( Peterson *et al*., 2014). Collectively, these elements are some of the key focus areas for operationalisation of the Transition and Sustainability Plan.

However, promoting community engagement is not necessarily an easy task, and developing and operationalising the community engagement plan is central to facilitating the engagement process ( Njue *et al*., 2015; Zulu *et al*., 2018; Zulu *et al*., 2019; Zulu *et al*., 2014a). In practice, this implies identification of opinion or community leaders, village or community champions and community members who truly represent community needs ( Pratt *et al*., 2015). It also implies unpacking what constitutes contextual power relations and interests as these shape community participation dynamics ( Molyneux & Bull, 2013; Njue *et al*., 2015; Tindana *et al*., 2006) . Community participation is key in developing good community engagement plans as it increases trust between developers of the engagement plans and communities ( Molyneux & Bull, 2013; Tindana *et al*., 2006). 

While community engagement plans may play an important role in promoting sustainability of VMMC and other services, there is inadequate documentation of the types and roles of stakeholders at community level including their power and interest as well as objectives and strategies. Consequently, this paper aims to contribute towards addressing this knowledge gap by documenting the development process of the community engagement plan in Zambia. The paper outlines the type of stakeholders available at community level and their roles and strategies in the transition/sustainability phase of the VMMC programme. The power and interest stakeholder analysis model was utilised to developing the strategy ( Brugha & Varvasovszky, 2000). Stakeholder analysis, and in particular the power and interest model, was adopted as it can help generate knowledge about the relevant actors or stakeholders, their agendas, interests, and the influence or resources, and how such information can be used to develop strategies for managing these stakeholders in programme implementation ( Brugha & Varvasovszky, 2000). Further, undertaking this was vital given the recommendation that future research should more thoroughly engage with community participation theory and recognise the power relations inherent in community participation ( George *et al*., 2015).

In this paper, power was defined by stakeholders during the interviews as having the authority and resources (such as financial and knowledge/skills) to influence, stimulate, encourage, or inspire the community or others to accept VMMC services. This definition of power is similar that one by Dalglish *et al* ( Dalglish *et al*., 2015) who categorised power into three dimensions namely political authority, financial resources and technical expertise. Interest refers to willingness and commitment to support and participate in the implementation process of VMMC including uptake of VMMC services. The Clinton Health Access Initiative (CHAI), with funding provided by the Bill & Melinda Gates Foundation (BMGF), supported the Zambian Ministry of Health in developing the community engagement plan for the sustainability phase of the VMMC response.

## Methods

### Study site

This study was conducted in three provinces of Zambia namely Lusaka, Copperbelt and Muchinga. The two latter areas were selected because one province had the highest VMMC uptake rate (Copperbelt province) while the other one had the lowest VMMC rates (Muchinga province). Lusaka province is the headquarters city had relatively high VMMC uptake rates. An earlier version of this article can be found on Research square ( https://doi.org/10.21203/rs.3.rs-295480/v1).

### The study design

A qualitative case study approach was used for developing the community engagement plan for the maintenance phase of VMMC (24). The case study methodology is an empirical approach that investigates contemporary phenomena within a real-life context, where the boundaries between phenomena and context are not clearly evident and in which multiple sources of evidence are used [28]. The case study approach was considered as appropriate for this study because the CHA strategy was developed within a complex context, which involved social interactions, and which was dependent on multiple local. The study adopted the consolidated criteria for reporting qualitative research (COREQ) checklist as sensitizing concepts in framing the study.

### Data collection and sampling strategy

Data collection was conducted from August 2012 to September 2022. We held meetings with national implementers of VMMC from Ministry of Health and CHAI to shortlist actors involved in the demand creation of VMMC in Zambia at community level. The district and health facility coordinators assisted research team to recruit eligible participants into the study. To be eligible- for the study, one needed to have been involved in the implementation of VMMC or should have accessed the services and or helped the child access services. Purposive sampling was used to select study participants who were appropriate given the study objectives and outcomes. Data were collected using Key Informant Interviews (KIIs) and Focus Group Discussions (FGDs) with community stakeholders such as chiefs, health centre committees, counsellors, headmen, teachers, health workers, community-based volunteers as well as parents/caregivers. A total of 35 FGDs consisting of at least 6 individuals were conducted (12 per province). A total of 35 KIIs were conducted (12 per province). Twelve interviews and FGDs were conducted per province in order to take into account a wide variety of stakeholders such as neighbourhood health committees (NHCs), community health workers (CHWs), traditional leaders, church leaders, health workers, teachers and parents. Transport refund compensation of K50 was provided to the participants. The Power/Interest Matrix stakeholder analysis tool by based on Newcombe (2003) mapping and ranking process. The paper aimed to outline the type of stakeholders available at community level, their roles, power, interest as well as strategies in the transition/sustainability phase of the VMMC programme.

The data collection process started with a review of key documents on VMMC in Zambia. These documents included published papers on VMMC, the Zambia VMMC National Operational Plan [2016–2020] ( MOH, 2016), and the 2019 Transition and Sustainability Plan for the Zambia VMMC Programme ( MoH, 2019). Given that the focus was on Zambia, we targeted the Ministry of Health website as the main source of published documents on VMMC ( https://www.moh.gov.zm). Additional documents were obtained from VMMC coordinating office at the Ministry of Health. No search terms were used as there are few published documents on VMMC in Zambia, and also because documentary review was the secondary source of data.

All the interviews were conducted with all the stakeholders in the district at various primary health care centres, locally called health facilities (clinics) or health posts and community settings (at traditional leaders’) separately by either the fourth (TFLM), fifth (JM), sixth (MK)and last authors (MPC), all of whom who had training and experience in qualitative research. The authors had diverse backgrounds and qualifications (in anthropology, medicine and public health) which helped in improving credibility of data. For authors involved in data collection, three researchers held at least a master’s in public health and one had a bachelor’s in demography and population studies. The lead Investigator had a PhD in anthropology and public health. Three researchers where female and nine were male. Some researchers had relationship with stakeholder’s established prior commencement the study which facilitated good rapport and trust with the community.

We conducted interviews using a semi structured interview. Respondents were asked to state their role of the community in VMMC, sustainability of community driven VMMC activities, who to be engaged during the transition/sustainability phase of the VMMC, nature of the reporting process and feedback process, and monitoring mechanism of the plan. The interview guide can be found as *Extended data* ( Chavula, 2022).

 Most of the interviews were conducted in local languages namely, Nyanja and Bemba as respondents were conversant with the language. Interviews conducted in local language were transcribed and translated in English (official language). An average interview lasted for about 1:55 hours. Interviews were recorded using a voice recorder. The notes were also taken by during the interviews, and these were extensively reviewed and discussed.

### Data analysis

The analysis and writing process of the engagement plans started with transcribing data from the audio interviews from the field and consultative/ stakeholder analysis meetings. Data was familiarised by multiple readings of the transcribed interviews while paying attention to patterns and occurrence. The code framework was generated by two researchers working on the study (MPC & JMC). The framework was revised several times after agreeing with the research team which themes represented the majority views. The code and transcripts were then exported into a qualitative software ( Nvivo plus 12, RRID:SCR_014802) for data management and analysis. Coding involved identifying a passage in the text or other data items, searching and identifying concepts and finding relations between them ( Gibbs, 2007). Four researchers were involved in coding data into respective themes (MPC, JM, MK & TM). A workshop was held in Kabwe district on 1 ^st^ November 2019 to validate the data with participants drawn from all the study sites. In addition, officials from the Ministry of Health Headquarters attended the validation workshop. 

### Ethics

Ethical approval to conduct the study was granted by the Excellence in Research Ethics and Science Converge (ERES), REF.No.2019-May-033 and the National Health Research Authority. Written and verbal informed consent was sought from all participants and high levels of confidentiality were observed throughout the stakeholder engagement process. To ensure confidentiality, the names of the respondents including any possible identifiers were removed during the report writing and dissemination process.

## Results

### Actors currently involved in VMMC in the community

The main actors at community level who were involved in VMMC activities included the neighbourhood health committees (NHCs), community health workers (CHWs), traditional leaders, church leaders and teachers. In Zambia, these actors operate as part of the community health system structured at the lowest level of service delivery and utilises health centres and health posts and make up the highest proportion of health facilities. These structures work together with different committees such as the Health Centre Committees (HCC) and NHCs to provide health services in the community. The NHCs also work with community leaders such as traditional leaders, religious leaders, and political leaders through Ward Development Committee (WDC).

### Power and interest of stakeholders in the transition/sustainability phase of the VMMC

**Figure 1.**
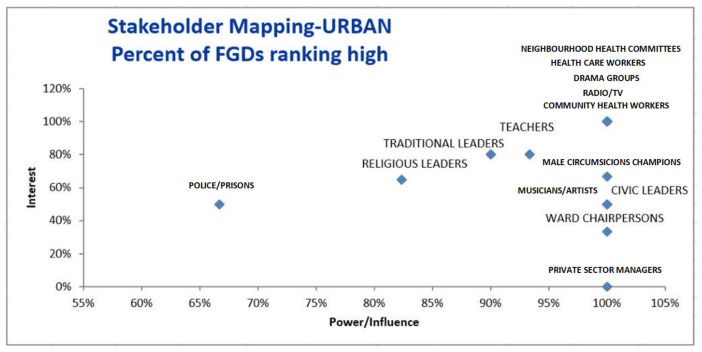
Stakeholder mapping – Urban sites. FDG=focus group discussion.

**Figure 2.**
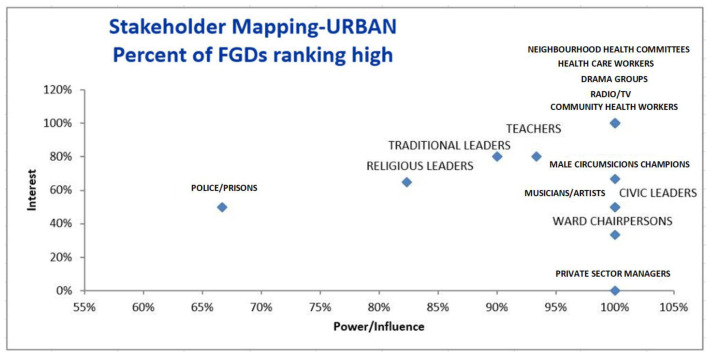
Stakeholder mapping – Rural sites. FDG=focus group discussion.

Data revealed that different stakeholders in urban and rural districts are of primary importance. Figure 1 and Figure 2 provide ranking of different stakeholders in terms of interest and power/influence. Stakeholders farther to the right have higher power/influence, and those farther up on the graph have higher interest, meaning that those stakeholders in the highest right quadrant have the most interest and power/influence according to the FGDs.

***The first category of stakeholders.*** In the urban setting, all FGDs ranked neighbourhood health committees (NHCs), health workers, drama groups, community health workers (CHWs) and radio/TVs as having high power/influence and interest ( Chavula, 2022). In the rural setting, all FGDs ranked club leaders, health workers and radio/TVs as having high power/influence and interest. Thus, from these rankings, drama groups were only rated as having high power/interest in the urban area while club leaders were only rated as having high power/interest in the rural settings. While all FGDs in the urban area ranked CHWs as having high power and interest, differences were noted in the rural area. Analysis of FGDs showed that rural areas ranked CHWs as having high interest while only about 85% rated them to have high power.

***The second category of stakeholders.*** The second category of stakeholders namely, teachers, traditional leaders, religious leaders- and male circumcision (MC) champions in the urban areas were rated to have high interest by around 60%– 80% percentage points. These stakeholders were also rated by more than 80% of the FGDs in urban sites as having high power. The second category of stakeholders also included youths, NHCs and parents in the rural areas. While the majority of the FGDs (100%) in the urban sites rated these stakeholders as having high power/influence, fewer FGDs (less than 60%) rated them as having high interest in VMMC.

***Third rank/ category of stakeholders.*** The third and lowest category of stakeholders included musicians/ artists, civic leaders, ward chairpersons and private sector managers. While the majority of the FGDs (100%) for example in the urban sites rated these stakeholders as having high power/influence, fewer FGDs (less than 60%) rated these stakeholders as having high interest in VMMC. These were rated as having high power because they usually have access to financial resources and that they lead or have many people who follow them. However, their interest in circumcision maybe low due to limited knowledge.

### Rationale for stakeholder rankings

This section explains how and why the respondents ranked stockholders being in the first or second, or third categories in terms of the sustainability phase of VMMC. Table 1 below provides a summary of the rankings of the stakeholders.

**Table 1.** Power stakeholder ranking. NHCs=neighbourhood health committees, CHWs=community health workers.

	First Rank (highest power + influence)	Second Rank	Third Rank (lowest power + influence)
**URBAN**	Health care workers, NHCs, CHWs, Radio/TV, Social Media, Club Leaders	Teachers, Religious Leaders, Parents	Musicians/ artists, civic leaders, ward chairpersons and private sector managers
**RURAL**	Health care workers, NHCs, CHWs, Drama, Radio	Teachers, Traditional Leaders, Religious Leaders, Parents	Famers, councillors, musicians/ artist, civic leaders

The main forms or sources of power that emerged in the interviews included technical expertise, local authority, financial resources, community health settings, and relational power. As shown in the table above, some stakeholders such as health workers, teachers, CHWs, traditional leaders, religious leaders had more sources of power than others like parents and private sector managers. Table 2 provides more details on the forms or sources of power. 

**Table 2.** Summary of sources/ forms of power. CHWs=community health workers.

Forms/sources of power	Description of forms/sources of powers	Types of stakeholders
Technical expertise	Knowledge, skills, roles	Health workers, CHWs, teachers
Local authority	Traditional, political, religious leadership	Traditional leaders, religious / church leaders, civic leaders (ward chairpersons and councillors)
Financial resources	Finances	Private sector managers, farmers
Collective action	Schools, churches, health facilities, media platforms, community spaces	Traditional leaders, religious / church leaders, civic leaders, musicians, club leaders, media platforms, youth champions, CHWs
Relational	Community / family bonds	Parents, care givers

### First rank (highest power + influence)

***Health care workers.*** The participants ranked health care providers with high power and interest because they have technical expertise to conduct medical circumcision. Further, health workers have a socially recognised setting or base from which they perform their roles. They were rated as having high interest because despite the shortages in human resources for health, they still attend to clients undergoing VMMC. Health care workers also work in collaboration with various structures or stakeholders such as traditional leaders, NHCs, CHWs, and religious leaders to create demand for VMMC.

*“The health professionals who are responsible in circumcising the people have a lot of interest – and power as their major task is to provide circumcision services”* (FGD, Community Based Volunteers, Chongwe District).

***Neighbourhood health committees and community health workers.*** It was noted that the NHCs and CHWs have power because they are knowledgeable/ skilled as they were trained to sensitise and refer people for circumcision *.* Furthermore, they also have recognised community spaces or settings from where they hold meetings to educate the people regarding circumcision. Being members of the community and having close relations/bonds with community members, they have an interest to ensure that their community is healthy.

 “ *The role of community health workers and NHC is that if they get information here at the clinic on circumcision - they take it to the community*. *They are the link between the community and health facility”* (FGD, NHC, Chongwe District).

***Radio/TV/ social media platforms.*** Radio/TV/ social media communication platforms were ranked high because they are important for community health settings as a means of disseminating health information about circumcision. Some stakeholders argued that although the media is extremely important in creating demand, radio and TV may not be accessible to all people especially to those living in rural areas. In addition, the language barrier is another challenge which might affect many others from accessing information because they may not understand the information. 

***Club leaders.*** Leaders of community groups such as clubs for women, sports and farmers were identified as other key groups that would be essential to involve during the transition phase for VMMC. The leaders are key because they have influence over their members. They further noted that use of these channels or community health settings would be compatible with the community practices as such groups have been previously used to communicate health messages in the community.

### Second rank

***Teachers.*** Teachers were viewed as powerful because they have formal platforms or community health settings (schools) for providing health education, including on VMMC. Teachers were further ranked to have high power and interest given the amount of time pupils spend at school than at home. In addition, teachers are also viewed by learners as role models and opinion leaders and can thus influence behaviour change for learners under their portfolio.

*“Teachers can spread the information in the community through school going children. As learners always look up to teachers, they can easily take up messages on circumcision once taught”* (FGD, Women, Chongwe District).

***Traditional leaders.*** Most participants indicated that traditional leaders had high power and interest because the community is more likely to listen or obey them as they are custodians of all the traditions, norms, and practices in the community. Further, traditional leaders have power interest because they hold community meetings in community spaces/ settings to provide health education. Power is also exercised through giving penalties (local authority) to those who do not adhere to instructions from the traditional leaders.

*“Headmen and chiefs are powerful, because when chiefs talk, people will have that fear, and thus follow as it will be considered as tradition which should be done. For example, in North-western province, circumcision is just a tradition which has to be done whether you like it or not as long as you are a man. They have powers because they have influence over people, - they can easily control people or have the powers to enforce an activity in the community”* (FGD, Young men, Mpika District). 

***Religious leaders.*** Several reasons guided the ranking of religious leaders. Zambia is considered a Christian nation, and as such respondents revealed that religious leaders (mostly Christians) are powerful as they able to educate their congregants on the benefits of men undergoing circumcision through the existing religious programmes. In addition, respondents indicated that religious leaders have a lot of influence and interest as they can use religious community settings (churches) and authority in the form of religious rituals on circumcision to sensitise the community on the importance of VMMC. 

*“The church has power because they have the examples from the Bible (on male circumcision), this helps them to encourage their members to access circumcision with authority*”(FGD, Community Members, Chinsali District).

***Youth champions.*** The rating of youth/ VMMC champions was based on the assessment that young people have opportunities to meet in safe spaces or community settings where they are able to discuss health related issues freely and should therefore be the key target for VMMC. Some communities that have engaged young people and made them understand the importance of VMMC have recorded increased involvement of young people in educating and influencing their fellow young people to access VMMC services. 

*“Some young people work as champions, so we visit those who are not yet circumcised and inform them on the benefits of VMMC, and once we have convinced them to participate in this programme, they also easily get other young people on board as young people easily attract the attention of other young people”* (FGD, Community Members, Chinsali District).

***Parents.*** Respondents reported that some parents have been supporting VMMC uptake. Particularly, this has been through decision making on behalf of their children. Thus, if a parent were informed, they would hold more influence over the decisions they make for their children, and that children usually listen to their parents. However, some participants argued that parents needed to be ranked lower than other stakeholders because parents are traditionally not comfortable to openly discuss issues of sexuality and VMMC with their children irrespective of whether it is in a rural or urban setup. 

*“At the moment, we even see more parents taking their children for circumcision and the number of children getting circumcised is more than other age groups. Furthermore, parents are even the ones who assent for their children to get circumcised, if they do not have interest for their children to get circumcised, they cannot bring them”* (FGD, CHWs, Lusaka).

### Third rank (lowest power + influence)

The third and lowest category of stakeholders included musicians/ artists, chairpersons ward (civic leaders), private sector managers and farmers. While the majority of the FGDs for example in the urban sites rated these stakeholders as having high power/influence, fewer FGDs (less than 60%) rated these stakeholders as having high interest in VMMC. These were rated as having high power because they usually have access to financial resources (managers and farmers), that they lead or have many people who follow them and have access to community health settings or spaces (musicians/ artists), and that they have political authority. However, their interest in circumcision maybe low due to limited knowledge.

 “ *Like musicians, they have a lot of influence in the community because people follow their music but may not be interested in circumcision activities because of lack of knowledge, thus if they can ---be sensitised on the importance of circumcision, then they can motivate many people who follow them to accept circumcision”* (FGD, Young Men, Chongwe District).

### Roles and strategies for strengthening community engagement in the transition/ sustainability phase of VMMC

This section presents the roles and strategies for facilitating community engagement in the transition/ sustainability of phase of VMMC. In each sub section, we begin by outlining the key role and then the strategies for achieving the role. 

### Integrating VMMC into primary health care

Integrating VMMC into primary health care was one of the roles that was mentioned in the interviews. Strategies for facilitating this role were making VMMC as part of the normal working routine at health facilities and building the capacity of different stakeholders in the community. 

***Making VMMC as part of normal working routine at health facilities.*** Developing strategies that would make VMMC health education a component of routine activities at health facilities was widely recommended. Study participants stated that integration is vital as it would make VMMC a point of discussion in almost all departments at health facilities, thus triggering improved appreciation and uptake of VMMC services. It was noted that integrating VMMC activities into ongoing community health outreach activities such as family planning talks, growth monitoring and promotion and under-five talks is sustainable as these activities do not require new funding.

*“I think like even integration with other services can help improve uptake of VMMC-- usually, we have the community members who work as volunteers, and when they are conducting like these other programmes in the area of maternal and child health, like community growth monitoring, I think they can also integrate VMMC – and this can be helpful”l* (KII, Sister in Charge. Kitwe District).

***Building the capacity of different stakeholders in the community.*** To facilitate service provision, it was suggested that capacity building of community members needs to be prioritised. Training various stakeholders such as volunteers, community leaders and NHCs on the importance of the VMMC, including how to conduct community mobilisation, skills in monitoring and evaluation and project management can make these stakeholders gain understanding, competencies, and courage to communicate and promote VMMC in the community. 

*“It’s important to involve community, by training a few community members so they can go in the field, and they can talk about it to the other community members. Maybe, if they see it come from their fellow community members, maybe they can even welcome it (circumcision)” (*KII, Female, Class teacher, Lufwanyama District).

### Participating in local VMMC planning processes

The main strategies for enabling stakeholders effectively to participate in planning VMMC included developing community VMMC steering committees, broadening spaces for citizen engagement, mobilising existing community resources and prioritising gender inclusion in planning of VMMC.

***Developing community VMMC steering committees and integrating them into existing committees*** Most respondents suggested that development of VMMC technical working groups to coordinate VMMC activities at community level and integrating them into existing community committees such as the NHCs is vital in promoting community participation, sustaining and scaling up VMMC. Formation of committees is key not only for coordination purposes but also co-production of VMMC services through collaborative planning processes. Involvement of community stakeholders in the planning process would help generate interest and subsequently power to drive VMMC activities at community level.

*“If we can involve the church and community leaders, no funds will be required to conduct the activities as they can also go out there to sensitise other people using their normal platforms” (*FGD, NHC, Members, Mpika District).

***Broadening spaces for citizen engagement in planning process.*** There is a need to create public forums or spaces through which the community can participate in planning VMMC activities to generate more interest and support from the traditional leaders, youths, religious leaders and parents towards VMMC. This was noted as important because currently not all community members, such as youths, are involved in decision making processes. To achieve this, there is need for increased sensitisation by NGOs on the relevance of community participation in decision making.

*“We need to have regular meetings, and in which many members of the community can meet to strategise and plan on how to proceed with activities on circumcision”* (KII, Male Religious Leader, Lusaka District).

### Mobilising of existing community resources

The other aspect of co-production was participation by the community in mobilising local resources. The respondents narrated that in case there is no funding from the Ministry of Health, the community can engage the private sector or businesses to disseminate printed information on VMMC to their customers through displaying such information in various shops and supermarkets. The community members cited examples of how in the past they have been able to contribute local resources towards infrastructure development such as construction of a maternity ward.

*“At some point we never used to have water in the maternity ward, and we did everything possible to make sure that we bring water to the ward. So based on our previous experience, we believe that we can work together and mobilise resources for circumcision from different sources”* (FGD, Community Based Volunteers, Lufwanyama District).

### Providing VMMC information using locally appropriate communication channels

Key strategies for enhancing the provision of VMMC information using locally appropriate communication channels included use of locally recognised communication spaces and channels such as the school system (integrated science subjects) and use of spaces managed by community leaders to promote uptake of VMMC.

***Integrating VMMC in the school system.*** Integrating VMMC into the schools was one of the suggested ways of facilitating increased participation of teachers in delivering and sustaining VMMC activities at community level. While schools have been used in some cases as platforms for sharing information on VMMC, it was noted that this has not been done in a standardised and comprehensive manner. One way of institutionalising VMMC within the school is through integrating VMMC into the school curriculum. To support the integration process, participants indicated that there is a need to train teachers in communicating information on VMMC. In addition to reaching out to a bigger audience within a short period, such integration would help in socialising young people at an early age. Further, integration in the school curriculum was viewed as effective as teachers are influential and highly respected in their communities. 

*“In schools they have to include male circumcision in the school curriculum because reproduction is there already, so even this one they should include it so that pupils can start learning about male circumcision”* (FGD, CHW/Vs, Kitwe District).

***Use of locally recognised communication spaces and channels.*** Stakeholders suggested that it was important to make use of the spaces and channels that are managed by community leaders such as traditional and religious leaders in disseminating information on VMMC. Such spaces include community meetings and traditional ceremonies. It was widely noted that traditional leaders have high influence in the community, and as such they had the power to impact VMMC activities positively. Traditional leaders, especially in rural areas, are usually the first contact persons that programme implementers meet or interact with before interacting with the wider community. They are seen to hold the key to high community participation in programmes.

*“We will be calling the parents to attend awareness meetings on circumcision, and for those parents that will show lack of interest in coming to meetings and taking the children for circumcision, we call them and make them answerable”* (KII, Male, Traditional Leader, Chinsali District).

It was further reported that involving traditional leaders would help the community view circumcision as a social phenomenon which is embedded in the local cultural practices. This was widely discussed as a few ethnic groupings in Zambia have male circumcision as part of their cultural practice. 

*“Some ethnic groups in Zambia have male circumcision in their traditions, and we can emphasise the cultural value; because if we link it so much to diseases, then it starts looking like it’s a medical issue”* (FGD, WDC Chairpersons, Kitwe District).

While religious leaders have been involved in promoting VMMC, not all are participating currently. The use of churches was cited as important because Zambia is Christian nation and thus religious values, and doctrines shape the decisions and behaviour of many people in the country. It was also noted that currently churches are also engaged in providing health information on various issues such as HIV/AIDS, drug and substance abuse and general health issues, to mention but a few.

*“We have to explain to them that it is custom of all Christians to be circumcised, and the scientific reason as to why it is like that is because of the dirty which is found in the foreskin which is later passed on to women who eventually get sick of cervical cancer”* (FGD, Community Men, Lufwanyama District).

***Integrating participatory approaches in VMMC health education.*** The community members suggested that it might be important to adopt and use more innovative approaches to health education. The main inventive approaches which were proposed are various media platforms, drama, and sporting activities. For such programmes to be locally relevant, it was noted that there is need for involvement of local people in developing health education including holding debates on VMMC. This can also include the development of local films which can be shown on local TV stations and plays aired on local radio stations.

*“So, when we have some TVs in the OPD, we can ask the patients as they wait to watch some film or video on the benefits of circumcision”* (KII, Sister in-charge, Female, Matero, Lusaka District).

Participants also recommended that more young people should be trained on how to integrate VMMC messages into drama (theatre). It was suggested that such approaches may help in reaching many people within a short period as drama performances may attract many people to come to one place. 

*“One way is to have at least a drama group which can be going out in the villages, as they do drama, people will come together, and then those in charge can now talk to the people about circumcision” **(*** KII, Female, Traditional Leader, Lufwanyama District).

### Promoting ownership of VMMC processes

The key strategy for promoting ownership of VMMC processes included strengthening participation of local actors local monitoring and accountability processes.

***Strengthening local monitoring and accountability processes.*** Most of the respondents reported that there is a need to integrate VMMC in the current monitoring and accountability processes. Strengthening VMMC community monitoring and accountability systems is important in documenting lessons learnt to inform the sustainability processes of VMMC programmes. Community based participatory evaluation is relevant as it can facilitate development of appropriate or locally relevant implementation processes since local communities lead the development process. Further, the participatory approach can, through iterative and inclusive dialogue at various levels of the research process, promote local capacity building geared towards a collective social change. The participants highlighted that there is a need to develop other tools to effectively capture community driven activities such as health education, distribution of information education counselling (IEC) materials, referrals, and community sensitisation.

 “ *It’s when we evaluate that you see and learn that it is true that this programme that we have started is working or not working. For circumcision, we need to make sure that the community is involved in capturing data on the progress of activities for promoting circumcision”* (KII, Male, Treasurer, NHC, Lusaka District).

*“As traditional leaders, we can also come up with stakeholder meetings and discuss issues about male circumcision including regularly checking on how these activities are being handled in the community and coming up with ways for improving uptake of circumcision services”* (KII, Male, Traditional Leader, Mpika District).

## Discussion

Analysis of types of stakeholders as well as roles and strategies that stakeholders can play in facilitating sustainability of reproductive health programmes such as VMMC services showed that different stakeholders have different power and interest in relation to VMMC services. Like Dalglish *et al*. ( Dalglish *et al*., 2015) who described three forms of power namely *political authority*, *financial resources* and *technical expertise* as key in implementing community health programs, this paper also documented similar forms, and two additional forms of power. In this study technical power included knowledge, skills, and roles that are performed by different stakeholders at community level. Financial power was about access to money to support implementation of VMMC including awareness activities. Meanwhile, political authority was defined slightly differently in this study as local authority which included traditional, political, religious leadership. In addition to these three forms of power, the study identified additional forms or sources of power namely *collective action* which consists of activities conducted in communal spaces such as schools, churches, health facilities, media platforms, as well as *relational power* which is about community / family connectedness. Mapping and considering these forms of power ( and in particular the two additional forms of power) when implementing programmes is vital as a community health system “ *consists of the set of local actors, relationships, and processes engaged in producing, advocating for, and supporting health in communities and households outside of, but existing in relationship to, formal health structures” ( Schneider & Lehmann, 2016; Schneider *et al*., 2022).*

This paper has also shown that in addition to differences in power among actors, there are also differences in interest among actors depending on their power, position and responsibilities in society. These diverse interests coupled with power differential could result in contestation or multiple agendas which could affect delivery, uptake and sustainability of health programmes including VMMC. Contestation is possible as communities are complex as they *involve a large number of diverse elements, that interact dynamically, often in non-linear ways, informed by direct and indirect feedback, in open systems with memory and adaptive capacities ( George *et al*., 2018)*. Thus, mapping of key stakeholders, including how diverse actors interpret issues (interest), respond (power) and adapt to (roles and strategies) programmes, is important if VMMC programmes are to be successfully implemented and sustained as implementation pathways of community programmes are more varied and more porous ( George *et al*., 2018; Zulu *et al*., 2015).

To effectively manage varied power and interests, there is a need to widen participation and promote collective action and accountability ( George *et al*., 2018; Mulubwa *et al*., 2020; Schneider *et al*., 2022; Tetu *et al*., 2021), in the delivery of VMMC. This study showed that increasing stakeholder engagement in planning, delivering, and monitoring VMMC programmes could be one way of enhancing the delivery, uptake, and sustainability of VMMC. Specifically, key sustainability strategies need to focus on integrating VMMC into primary health care, strengthening local VMMC planning processes, adoption of locally appropriate communication channels for delivering VMMC messages as well as promoting local ownership of VMMC processes.

Studies have shown that several stakeholders at community level such as religious and traditional leaders, traditional healers, health care workers, neighbourhood health committees and other community members shape the uptake of VMMC services in the community ( Bulled & Green, 2016; Mbonye *et al*., 2016). Traditional and religious leaders are helpful in providing support for an approach that takes into account local beliefs about circumcision ( Bulled & Green, 2016; Mbonye *et al*., 2016). Community engagement further provides opportunities for collaboration with traditional and cultural sectors to enhance understanding of the correct messaging on VMMC that address topics such as manhood and masculinity, gender norms, sexual consent and readiness for sex ( WHO, 2007). Involvement of local leadership also increases public enthusiasm about HIV prevention programmes. Furthermore, engagement helps in ensuring that health facility leadership work with community leaders and teachers to determine site-specific VMMC services which are complemented by occasional community-based campaigns ( Chavula *et al*., 2021; Feldacker *et al*., 2018). Such processes can result into integration of VMMC into the health system as well as ownership and sustainability of VMMC by community members ( Bailey *et al*., 2008).

Buckley *et al*. (2014) suggests that an integrated or blended approach which involves local participation may not only improve uptake or ownership but also promote sustainability by strengthening local health systems ( Bailey *et al*., 2008; Zulu *et al*., 2021). Engagement could be useful in promoting sustained acceptability and support towards health processes by “building authentic partnerships, including mutual respect and active inclusive participation; power sharing and equity; mutual benefit or finding the ‘win–win’ possibility in the collaborative initiative”( Tindana *et al*., 2006). Community engagement is important as community values, beliefs and norms tend to influence views on risks and benefits of health programmes and research, thus affecting independent decision making or consent process ( Pratt *et al*., 2015; Tindana *et al*., 2006; Tindana *et al*., 2015). Additionally, engagement of stakeholders helps in ensuring progress towards greater local accountability, quality improvement, and compliance with safety standards in a manner that encourages sustainability of these efforts ( Feldacker *et al*., 2018; Zulu *et al*., 2014b).

### Strengths and limitations

The collection of data from various stakeholders involved in the creation of VMMC programme enabled us to gather rich information on the subject. This also helped us to gain a better understating of themes from different perspectives and sources. Furthermore, the research team comprised professionals from various academic backgrounds such us public health, social sciences and health sciences, which could have shaped the analysis and interpretation of results. However, there were also limitations to this study. We conducted this study in three provinces in Zambia. The results may not be a representative of the whole country perspectives, due to cultural and societal perceptions, values and norms around VMMC. We however attempted to address this limitation by including both rural and urban sites in the six districts that where sampled. The prolonged engagement and interaction between participants and researchers helped to gain a deeper understanding of the subject. 

## Conclusion

There were differences between the rural and urban sites in terms of power and interest rating of stakeholders who could be involved in the sustainability phase of the VMMC response in Zambia. Five forms or sources of power namely technical expertise, local authority, financial resources, community settings and relational power were documented. Roles and strategies for strengthening community engagement in the sustainability phase of VMMC included integrating VMMC into primary health care through making VMMC part of normal work schedule at health facilities and building the capacity of different stakeholders in providing VMMC, participating in local VMMC planning processes by developing community VMMC steering committees and integrating them into existing committees, broadening spaces for citizen engagement in planning process and mobilisation of existing community resources. Additional roles and strategies were providing VMMC information using locally appropriate communication channels and promoting ownership of VMMC processes through strengthening local monitoring and accountability processes. Community participation in documenting power and interest among stakeholders as well as roles and strategies of stakeholders in delivering VMMC may facilitate development of VMMC sustainability plans that are accepted and owned by the community.

## Data availability

### Underlying data

Fighshare: Transcripts- Community engagement for the Voluntary Medical Male Circumcision program an analysis of key stakeholder roles to promote a sustainable program in Zambia.zip. https://doi.org/10.6084/m9.figshare.19494254.v2 ( Chavula, 2022).

This project contains the following underlying data:

- Transcripts-Community engagement for the Voluntary Medical Male Circumcision program an analysis of key stakeholder roles to promote a sustainable program in Zambia.zip

### Extended data

Fighshare: Transcripts- Community engagement for the Voluntary Medical Male Circumcision program an analysis of key stakeholder roles to promote a sustainable program in Zambia.zip. https://doi.org/10.6084/m9.figshare.19494254.v2

This project contains the following extended data:

- Final tools CHAI VMMC tools-Community engagement for the Voluntary Medical Male Circumcision program an analysis of key stakeholder roles to promote a sustainable program in Zambia.docx (interview guide)

Data are available under the terms of the Creative Commons Zero "No rights reserved" data waiver (CC0 1.0 Public domain dedication).
