# Supplementary material for: Community engagement for the Voluntary Medical Male Circumcision (VMMC) program: an analysis of key stakeholder roles to promote a sustainable program in Zambia
Source: Gates Open Res. 2023 May 26;6:50. Originally published 2022 Apr 22. [Version 2] doi: 10.12688/gatesopenres.13587.2 (PMC10105033; doi:10.12688/gatesopenres.13587.2)
Supplement: Peer review history

# Peer review: Reviewer response for version 2

**DOI:** 10.21956/gatesopenres.16030.r33443

**Published:** 2023-05-31

**Authors:** Pammla Petrucka

**Affiliations:** 1 Aga Khan University, Karachi, Pakistan; 2 College of Nursing, University of Saskatchewan, Saskatoon, SK, Canada

Thank you for the extensive consideration of the comments and suggestions by reviewers. The table is helpful and summative.

Is the work clearly and accurately presented and does it cite the current literature?

Partly

If applicable, is the statistical analysis and its interpretation appropriate?

Yes

Are all the source data underlying the results available to ensure full reproducibility?

Yes

Is the study design appropriate and is the work technically sound?

Yes

Are the conclusions drawn adequately supported by the results?

Yes

Are sufficient details of methods and analysis provided to allow replication by others?

Partly

Reviewer Expertise:

Global Health, Women's Health in Development

I confirm that I have read this submission and believe that I have an appropriate level of expertise to confirm that it is of an acceptable scientific standard.

# Peer review: Reviewer response for version 1

**DOI:** 10.21956/gatesopenres.14857.r32893

**Published:** 2023-04-14

**Authors:** Claudia Abreu Lopes

**Affiliations:** 1 International Institute for Global Health, United Nations University, Kuala Lumpur, Malaysia

The objective of this article is to outline the type of stakeholders in rural and urban communities, their roles, power, interest and strategies in the transition/sustainability phase of the Voluntary Medical Male Circumcision (VMMC) programme in six districts of Zambia. The qualitative study involved a desk review, Key Informant Interviews (KIIs) and Focus Group Discussions (FGDs) with community stakeholders such as chiefs, health centre committees, counsellors, headmen, teachers, health workers, community-based volunteers and parents/caregivers. The study is well structured with important insights from the stakeholder analysis that ranked stakeholders based on their relative importance in terms of interest and power. The differences between rural and urban communities are underlined and the ranks are explained based on sound evidence from the FGDs. The contribution of this study is the recommendation of sustainability strategies for VMMC, informed by the stakeholder analysis and the KIIs, that can be applied to other community health interventions in Zambia.

 Notes: 

- Figure 1 and 2 are the same.
- In the introduction para 3, this idea appears twice: VMMC was recommended by WHO as a high-impact cost-effective intervention, particularly in settings with high HIV prevalence and low levels of male circumcision.

Is the work clearly and accurately presented and does it cite the current literature?

Yes

If applicable, is the statistical analysis and its interpretation appropriate?

Not applicable

Are all the source data underlying the results available to ensure full reproducibility?

Yes

Is the study design appropriate and is the work technically sound?

Yes

Are the conclusions drawn adequately supported by the results?

Yes

Are sufficient details of methods and analysis provided to allow replication by others?

Yes

Reviewer Expertise:

Global health: community engagement, implementation research, gender and health, digital health

I confirm that I have read this submission and believe that I have an appropriate level of expertise to confirm that it is of an acceptable scientific standard.

# Peer review: Reviewer response for version 1

**DOI:** 10.21956/gatesopenres.14857.r32892

**Published:** 2023-02-22

**Authors:** Pammla Petrucka

**Affiliations:** 1 Aga Khan University, Karachi, Pakistan; 2 College of Nursing, University of Saskatchewan, Saskatoon, SK, Canada

The article speaks to a critical aspect of scaling up VMMC services as a critical HIV prevention (mitigation) measure – specifically speaks to the imperative of engaging communities especially in shifting culturally ascribed to beliefs respecting non-circumcision. Mobilizing for participation, change, and sustainability are critical – and potentially achievable with consultation and involvement of a diverse community-based stakeholder profile.

 The qualitative case study approach enabled utilization of a range of data sources (i.e., KIIs,and FDs) to unveil a complex issue. The COREQ framework was highly appropriate.

 The connection of the stakeholders’ roles in the VMMC success was situated in the theoretical and contextual realities.

**Study strengths:**

 The authors provide a concise and well-developed discussion of the relationship between circumcision and HIV rates as well as the communities’ power on the issue. The authors are commended for how they framed this issue and invoked a study design that supports PAR and sustainable interventions. The study design, data collection, analysis and discussion are well articulated. It is noteworthy that they have an inclusive approach. The categories of stakeholders along with the power/influence discussions were excellent.

**Study limitations:**

 The authors must reconsider the limitations of this study – it is important that they reflect on the elements of the design and sampling which may lead to limitations of this work. The study is well situated with the extant literature; however nearly 40% are from 2015 or before calling into concern the currency of the evidentiary base of this paper. A table or a diagram of the sampling and data collection methods would have strengthened this section – it was difficult to follow the narrative and interpret the reach. I would recommend additional clarity as to the validation workshops – what measures were taken for representativeness? Were there concerns about the presence of Ministry representatives in terms of power and/or integrity of the process especially in terms of confidentiality as these were participants being asked to respond or reflect on the findings. Perhaps there are missed opportunities to consider the cultural (tribal) issues, but this topic may have been outside the intent of the authors.

**Edits to be considered:**

**General:**

1. The manuscript requires another comprehensive round of editing to catch the grammar, sentence structure and syntax issues throughout.

**Introduction:**

1. The third paragraph under the introduction: should say “There were..” not “They were”.
2. Paragraph 5: the following is missing a comma for these targeted **populations,** as well as the general population, ….
3. Further down paragraph 5: this sentence is awkwardly worded and I suggest rewriting “However, implementation of the Transition and Sustainability Plan has **been limited so far, and in part due to** the lack of a community engagement plan."
4. Further down paragraph 5: VMMC becomes a norm at **THE **community level.
5. Paragraph 7: same as above for line 10.
6. Paragraph 7: “definition of power is similar that one by **TO** Dalglish *et al”.*

**Study Design:**

1. Under the “the study design” heading there is an incomplete sentence, “which was dependent on multiple local”.

**Ethics Issue: **

1. I suggest clarifying the dates for data conduction – 2012-2019 is stated but no clarification for this range when ethical approval wasn’t received until 2019.

Is the work clearly and accurately presented and does it cite the current literature?

Partly

If applicable, is the statistical analysis and its interpretation appropriate?

Yes

Are all the source data underlying the results available to ensure full reproducibility?

Yes

Is the study design appropriate and is the work technically sound?

Yes

Are the conclusions drawn adequately supported by the results?

Yes

Are sufficient details of methods and analysis provided to allow replication by others?

Partly

Reviewer Expertise:

Global Health, Women's Health in Development

I confirm that I have read this submission and believe that I have an appropriate level of expertise to confirm that it is of an acceptable scientific standard, however I have significant reservations, as outlined above.

# Peer review: Reviewer response for version 1

**DOI:** 10.21956/gatesopenres.14857.r32772

**Published:** 2022-12-15

**Authors:** Rodney Masese, Patrick Mapulanga

**Affiliations:** 1 Department of Community Health, University of Malawi, Kamuzu University of Health Sciences, Lilongwe, Malawi; 2 Kamuzu University of Health Sciences, Blantyre, Malawi

Malizgani Paul Chavula’s article titled *Community engagement for the Voluntary Medical Male*

*Circumcision (VMMC) program: an analysis of key stakeholder roles to promote a sustainable program in Zambia *is an original article that unearths a very crucial role key stakeholders can play in engaging the community for scaling up and sustaining VMMC services, particularly in non-circumcising communities. The World Health Organisation and UNAIDS recommended an increase in the rates of VMMC in low circumcision prevalence areas in order to end the HIV pandemic by 2030. However, promoting community participation as well as building the capacity of local NGOs is crucial not only for mobilising resources for VMMC service delivery, but also ensuring sustainability of the program. Therefore this paper addresses these pertinent issues by critically examining the types and roles of community stakeholders utilising scientifically-proven stakeholder analysis model.

 Through the power and interest stakeholder analysis, the paper has found that there are a variety of main community-based actors who must be closely engaged with to increase VMMC uptake. The study underscores the long-term cost-effectiveness and sustainability of VMMC services within non-circumcising communities if appropriate key stakeholders are meaningfully consulted and engaged.

 The diversity of the study sites, as well as the chosen methodology adds to the strength of the study. Drawing the views of the stakeholders from rural and urban areas broadens their representative views. In addition, the use of the qualitative approach in general, and the consolidated criteria for reporting qualitative research (COREQ) checklist in particular, provided an in-depth understanding of the power and interest of the key stakeholders in influencing uptake and sustainability of VMMC services at community level. More importantly, by using relevant analysis for qualitative data, the study has uncovered different roles and strategies for facilitating community engagement to make VMMC part of the normal working routine at health facilities in the community.

 Many of the references used in the study are current, though there are a few older literature that have been used because of their historical bearing on VMMC as a clinically-proven HIV prevention strategy. On the other hand, the study design is appropriate and makes the author’s work technically sound, with appropriate methods and analysis provided to allow replication of the study by others. The research article also contains rich data and discussion which is scientifically sound. In addition to logical conclusions supported by the results, data sources underlying the results have been made available for full reproducibility.

 Although the quality of the article is acceptable, there are a number of small changes that need to be considered to increase its scientific standard. Firstly, there are a few editorial issues which need to be looked into and edited. On page 4, under “Data collection and sampling strategy”, the first statement which indicates that data collection was from Year 2012 August must be verified in lieu of ethical approval granted in May, 2019. The other corrections include: Last but one sentence needs editing: “….analysis tool by…..” On page 5, second paragraph “Three researchers ***where*** females….” Third paragraph: “We conducted….interviews….” add ***guide***. Paragraph 4: “The notes were taken by….” (sounds incomplete).

 Lastly, given the complexity of the study and the varied sites where numerous stakeholders were engaged, only one limitation for the study sounds impractical. The authors also needed to explicitly suggest areas for further research. **All in all, in spite of the aforementioned small observations to the article, I believe that this submission is of an acceptable scientific standard worth my approval after those necessary changes.**

Is the work clearly and accurately presented and does it cite the current literature?

Yes

If applicable, is the statistical analysis and its interpretation appropriate?

Yes

Are all the source data underlying the results available to ensure full reproducibility?

Yes

Is the study design appropriate and is the work technically sound?

Yes

Are the conclusions drawn adequately supported by the results?

Yes

Are sufficient details of methods and analysis provided to allow replication by others?

Yes

Reviewer Expertise:

NA

We confirm that we have read this submission and believe that we have an appropriate level of expertise to confirm that it is of an acceptable scientific standard, however we have significant reservations, as outlined above.
